# Letters from Far Japan

**Published:** 1891-12-26

**Authors:** Ernest Hart


### LETTERS FROM FAR JAPAN.

By MRS. ERNEST HART.

(*Concluded from page 124*).

A FEW facts as to the cost of maintenance and the food of the prisoners will be interesting. I will give the figures in Japanese coinage. The average cost of the maintenance of a prisoner for a year is 59.80 dols. (£5 10s.) His food costs 4 sen 8 rin, about 1¾d., and his clothes 3 sen, about 1¼d. per day. His average earnings are 2 sen 6 rin (about one penny) per day. The prisons are supported out of the local rates, but the salaries of certain high officials are paid by the State. The prison diet is composed of rice and barley, with a relish of vegetables and pickles. The amount served out to each prisoner depends on the amount of physical labour done. Food is provided three times a day, and, as is always the case even in the poorest families in Japan, it is served neatly and daintily in bowls.

The officials of this prison expressed their desire to do away with this old wooden Tokugawa prison, and to construct a new brick building on more modern principles, but it seemed to me that, considering they had the space necessary, nothing could be more desirable than the system adopted here of separate open sheds, and that the healthful, cheerful conditions under which the prisoners work cannot fail to have a beneficial influence in their morals and intelligence.

Whether making prisoners happy has a deterrent effect in after life can only be estimated by experience and carefully kept records. The following figures, taken from the prison books, may throw a little light on the subject when compared with a similar set of figures taken from the records of an English prison :—

| | | Men. | Women. |
|---|---|---|---|
| Prisoners committed | for first offence ... | 818 | 80 |
| ,, ,, | for second offence ... | 340 | 30 |
| ,, ,, | for third offence ... | 384 | 9 |
| ,, ,, | for one month ... | 41 | 12 |
| ,, ,, | from one month to one year ... ... | 803 | 37 |
| ,, ,, | from one to five years | 462 | 19 |
| ,, ,, | from five to ten years | 27 | 19 |
| ,, ,, | from ten to fifteen years ... ... | — | 10 |

These figures seem to show that men are the more hardened criminals, and women the greatest, when their passions stir them up to commit crime, as in the case of the beautiful prisoner, Hai Ume.

Who knows who is to blame? "*Qui sait tout pardonne tout?*" and He alone who knows what caused the warp in the wood can trace crime to its source. Meanwhile the Japanese doubtless take the wisest course in bidding the criminal not despair of himself, to keep his self-respect, to still feel some enjoyment of the light and air and sunshine, and in freedom of movement though prison-bound, and to give him back his liberty, trained by industry and discipline to better run the race of life, in which the victory is with the strongest and best.

